# How a renal artery aneurysm may mimic renal stone?

**DOI:** 10.1016/j.eucr.2021.101621

**Published:** 2021-02-26

**Authors:** Abdolreza Mohammadi, Mohammad Reza Nikoobakht, Hamed Akhavizadegan, Seyed Mohammad Kazem Aghamir, Seyed Hassan Inanloo, Farnoud Nosrati

**Affiliations:** Urology Research Center, Tehran University of Medical Sciences, Tehran, Iran

**Keywords:** Renal artery aneurysm, Nephrolithiasis, Percutaneous nephrolithotomy, Endovascular embolization

## Abstract

Renal artery aneurysm is a rare condition. Most patients are asymptomatic. This case presented to the emergency with colicky pain and underwent left transurethral lithotripsy for left ureteral stone, due to simultaneous renal stone candidates for percutaneous nephrolithotomy. In the review of the imaging, we found a large renal artery aneurysm, so vascular surgery consult was done and the patient managed with coil embolization Misdiagnosis of this condition could be led to life-threatening bleeding if percutaneous nephrolithotomy was done. It is recommended that renal artery aneurysm should be considered in the differential diagnosis of renal rim-like opaque lesions.

## Introduction

Renal artery aneurysm is a rare condition.^1^ Most patients are asymptomatic and do not need intervention. This case presented to the emergency ward with colicky pain and underwent left transurethral lithotripsy for left ureteral stone, due to simultaneous renal stone candidate for percutaneous nephrolithotomy. In the review of the imaging study, we found a large renal artery aneurysm, so vascular surgery consult was done and the patient managed with coil embolization. Misdiagnosis of this condition could be led to life-threatening bleeding if shock wave lithotripsy or percutaneous nephrolithotomy was done. It is recommended that renal artery aneurysms should be considered in the differential diagnosis of renal rim-like opaque lesions.

## Case presentation

**Fig. 1:**
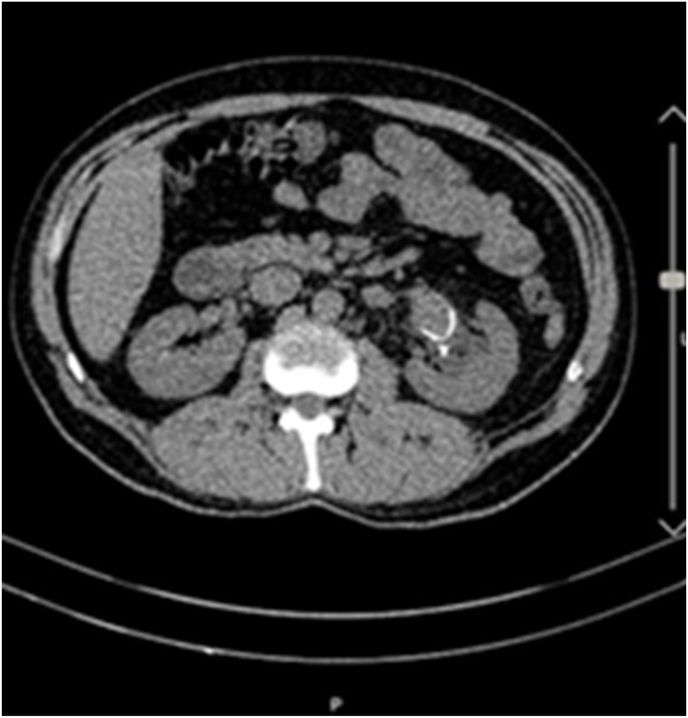
Left renal artery aneurysm.

**Fig. 2:**
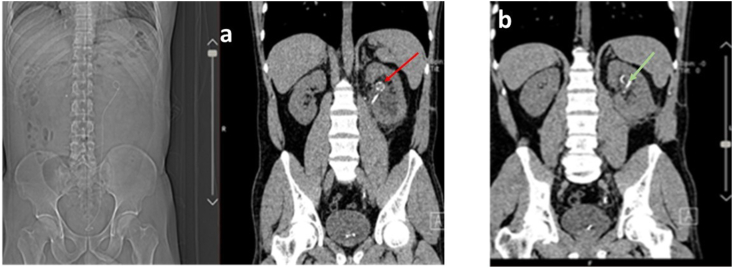
**a**: aneurysm (resemble Dj Stent head), **b**: Dj stent head.

**Fig. 3:**
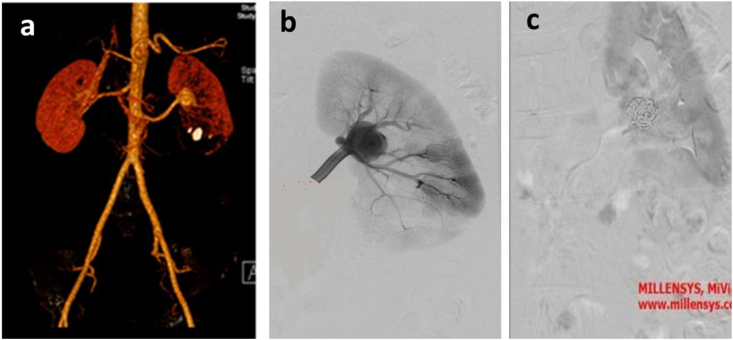
**a**: hilar renal aneurysm,**b**: pre-embolization image, **c**: post-embolization image.

A 44-year-old man was referred with left flank pain and medical history of renal stone; the only symptom was colicky pain for 5 days. In physical examination, he had left flank tenderness. At the time of initial presentation, urine analysis revealed 5–6 RBC but other laboratories finding such as CBC.diff, Urea, Creatinine, sodium, and potassium were in the normal range. Initial imaging with ultrasonography revealed a 19 mm left lower pole stone and an 11mm ureteral stone. He managed with Left transurethral lithotripsy and double j stent insertion. The patient was scheduled for left percutaneous nephrolithotomy (PCNL) but because of the stone size: 20 mm in the lower pole of the kidney, we reviewed the imaging study and we found a suspicious mass-like lesion with a rim of calcification in the left hilar area (Fig. 1) with a misleading appearance, very similar to Dj's head in some frames (Fig. 2a and b). Because of the renal artery aneurysm (RAA) peossibillity, the Computed tomography angiography (CTA) was performed (Fig. 3a) and revealed a 3cm left renal artery aneurysm. The PCNL was postponed and vascular surgery consultation was done due to flank pain and hematuria.

The endovascular intervention was performed through the femoral artery; stent insertion was tried but due to the inability for cannulating the lumen and complex anatomy of the aneurysm, embolization with three coils was done instead of the stent insertion (Fig. 3b and c). The patient was discharged as hematuria and pain resolved and he scheduled for future left PCNL. Post embolization ultrasound revealed normal blood flow in the kidney and the disappearance of the aneurysm.

## Discussion

Renal artery aneurysm is a rare condition with female predominance, the peak of incidence is 30–50 years. The natural history is benign and the growth rate is very slow in many cases.^2^ In imaging, incidental finding of the aneurysm is common, but it may be similar to the stone especially when ultrasound is performed as an initial imaging modality. In some cases, rim-shape calcification of the aneurysm may be led to misdiagnosis of stone so therapeutic modalities like SWL or PCNL could result in life-threatening bleeding.

Most cases are asymptomatic and discovered incidentally; due to the slow growth rate, there is no need for intervention in many patients. Indications for intervention that related to the size of the aneurysm is controversial but many experts agreed with >2 cm size whereas recently some expert mentioned that size of more than 2 cm is the aggressive approach. Other indications for intervention are women at childbearing age, pregnant women, uncontrolled hypertension, and symptomatic cases (pain and hematuria).^3^^,^^4^ In rare conditions such as multiple aneurysms and failed endovascular intervention, open surgery may be needed.^5^ In this case, due to the size of the aneurysm, pain, and hematuria, he was referred to a vascular surgeon and managed with coil embolization through femoral access; with improvement in symptoms and optimal renal blood supply in control imaging, he was discharged for future treatment, by the improvement of symptom and disappearance of the aneurysm in post embolization sonography, ureteroscopy, and laser lithotripsy as the first modality and PCNL due to stone size were proposed to the patient.

## Conclusion

Finally, we can say ultrasonography is not sufficient for stone diagnosis when modalities like PCNL and SWL are going to be performed only upon renal ultrasound, life-threatening bleeding of renal artery aneurysm will be possible. It's better to perform precise imaging in all patients with renal stones located in the hilum and renal artery aneurysm should be considered in the differential diagnosis of renal rim-like opaque lesions. It seems that in this case flexible ureteroscopy with laser lithotripsy may better option so we offered this modality to the patient.

## Declaration of competing interest

The authors declare that they have no competing interests.

## Abbreviations

CT: Computed tomography
CTA: Computed tomography angiography
DJ: Double J
PCNL: Percutaneous nephrolithotomy
RAA: Renal artery aneurysm
SWL: Shock wave lithotripsy
